# Ring-like (Donut-Shaped) Intracranial Aneurysms: A Warning Morphology of Mural Jet Flow and Pre-Rupture Instability

**DOI:** 10.3390/diagnostics16010078

**Published:** 2025-12-25

**Authors:** Dragoslav Nestorović, Andrija Savić, Petar Milenković, Miloš Stojaković, Tamara Švabić, Igor Nikolić

**Affiliations:** 1 Center for Radiology, University Clinical Centre of Serbia, 11000 Belgrade, Serbia; 2 Neurosurgery Clinic, University Clinical Center of Serbia, Faculty of Medicine, University of Belgrade, 11000 Belgrade, Serbia; 3 Ars Medica, 11000 Belgrade, Serbia; 4 National Cancer Research Center, 11000 Belgrade, Serbia; 5 Neurology Clinic, University Clinical Centre of Serbia, Faculty of Medicine, University of Belgrade, 11000 Belgrade, Serbia

**Keywords:** intracranial aneurysm, donut shape, ring-like

## Abstract

**Background/Objectives:** “Ring-like” intracranial aneurysms—historically described as “*doughnut-like*” or “*donut sign*”—represent a rare configuration in which a central thrombus coexists with a circumferential mural flow ring. Traditionally considered a radiologic curiosity, this morphology likely reflects a shear-driven hemodynamic state rather than a stable organized thrombus. We aimed to summarize all PubMed-documented cases of ring-like aneurysms, define their morphologic and clinical spectrum, and assess their hemodynamic significance, rupture risk, and treatment outcomes. An additional aim is to formalize the use of the term “ring-like aneurysm” as a distinct morphologic subtype and to clearly differentiate it from the neuroradiologic “donut sign,” which represents an imaging appearance rather than a specific anatomic configuration. **Methods:** A systematic PubMed search (1996–2024) was conducted using the following combinations of keywords and Boolean operators: (“ring-like aneurysm” OR “donut aneurysm” OR “doughnut aneurysm” OR “ring-shaped aneurysm” OR “circumferential lumen” OR “central thrombus”) AND (“intracranial” OR “cerebral” OR “basilar” OR “aneurysm”). Only English-language, PubMed-indexed reports describing true ring-like (donut-shaped) aneurysms were included. Non-indexed, non-English, and serpentine or fusiform aneurysms mimicking ring-like morphology were excluded. Extracted data included aneurysm location, size, presentation (ruptured, symptomatic, or incidental), treatment strategy, and clinical outcome. Statistical proportions were analyzed using descriptive methods, Wilson 95% confidence intervals, and a binomial test to compare the observed subarachnoid hemorrhage (SAH) rate against the expected conservative rupture proportion. **Results:** The search identified 16 individual patients reported in 10 publications. All aneurysms were large or giant (14–36 mm) displaying characteristic thrombosed pattern. Ruptured presentation occurred in 6 out of 16 cases (37.5%) and symptomatic unruptured in 10 (62.5%). No incidental cases were reported. Posterior circulation involvement was present in 44%, with a female predominance of 69%. **Conclusions:** Ring-like aneurysms constitute a distinct, shear-maintained hemodynamic entity combining mural jet flow with central thrombosis. Their frequent symptomatic or ruptured presentation supports the concept that this morphology represents a pre-ruptural configuration rather than a chronic thrombotic residue. Early recognition and targeted endovascular exclusion of the inflow zone are essential to prevent delayed rupture.

## 1. Introduction

The term “*doughnut-like*” aneurysm was first introduced by Ogawa et al. in 1996 [1], who described a partially thrombosed basilar tip aneurysm with a concentric flow lumen and central thrombus on 3-dimensional computed tomography angiography (3D-CTA) and digital subtraction angiography (DSA). This was the earliest recognition of the ring-shaped morphology—circumferential mural patency surrounding a central clot—that would later be referred to as the “donut sign” by van Rooij et al. eighteen years later [2]. Its reintroduction and subsequent popularization established the “donut sign” as a distinct imaging descriptor of partially thrombosed aneurysms. However, the “donut sign” should not be conflated with the ring-like aneurysm morphologic type. The “donut sign” denotes an imaging appearance that may also be observed in certain serpentine aneurysms [3] whereas the term “ring-like aneurysm” refers to a specific structural configuration. Despite its distinctive appearance, the morphologic boundaries and clinical significance of this configuration have not been clearly defined in the existing literature.

To address this ambiguity, the present study adopts the term *ring-like aneurysm* to denote an intracranial aneurysm with a circumferential residual lumen surrounding a central organized thrombus, in which inflow and outflow share a common neck. The objective is to consolidate all PubMed-documented cases, define the morphologic and clinical spectrum of ring-like aneurysms, and distinguish this entity from the neuroradiologic “donut sign.”

## 2. Materials and Methods

A systematic search of the PubMed database was performed for the period 1996–2024. The search used the following keyword combinations and Boolean operators: (“*ring-like aneurysm*” *OR* “*donut aneurysm*” *OR* “*doughnut aneurysm*” *OR* “*ring-shaped aneurysm*” *OR* “*circumferential lumen*” *OR* “*central thrombus*”) *AND* (“*intracranial*” *OR* “*cerebral*” *OR* “*basilar*” *OR* “*aneurysm*”). Reference lists of eligible publications were additionally screened to identify any secondary citations meeting inclusion criteria.

We included only PubMed-indexed, English-language reports that described true ring-like (donut-shaped) intracranial aneurysms characterized by a peripheral circumferential flow lumen surrounding a central thrombus. We excluded (1) non-indexed or non-English publications, (2) serpentine, fusiform, dolichoectatic, or partially thrombosed aneurysms that merely mimicked a ring-like configuration, and (3) reports lacking sufficient imaging or narrative detail to confirm a ring-like morphology.

For all included cases, we systematically extracted aneurysm location, maximal sac dimensions, clinical presentation (ruptured, symptomatic unruptured, or incidental), treatment modality (coiling, clipping, flow diversion, bypass, or conservative) and clinical outcomes. All data were independently verified by cross-referencing narrative descriptions with published imaging.

Descriptive statistics were used to summarize proportions across the pooled cohort. Rupture prevalence (SAH/ICH) and other categorical proportions were reported with Wilson 95% confidence intervals. A binomial test was used to compare the observed rupture prevalence with a conservative expected annual rupture risk derived from population-level data on large and giant intracranial aneurysms.

## 3. Results

Across the pooled cohort of 16 patients, 37.5% (6/16) presented with rupture (SAH or ICH), with a 95% confidence interval (CI) of 18.5–61.4%. Non-ruptured but symptomatic presentation accounted for 62.5% (11/16), corresponding to a 95% CI of 38.6–81.5%. All aneurysms in the series were large or giant (16/16; 100%), with a minimum sac diameter of ≥14 mm. A female predominance was observed (11/16; 69%), yielding an approximate female-to-male ratio of 2.2:1. Posterior circulation involvement was identified in 44% (7/16) of cases, with the basilar tip representing the most frequent location. Mortality analysis was performed on 15 evaluable patients, excluding the index case described by Ogawa et al. due to missing follow-up information. The overall mortality was 20% (3/15), with a 95% CI of 7.0–45.2%.

All publication cases were displayed in Table 1 chronologically.

## 4. Discussion

Although visually distinctive, the biological and hemodynamic significance of the ring-like configuration has remained insufficiently characterized. Subsequent reports have suggested that this morphology represents an active, shear-driven state rather than a stable, chronically organized thrombus [12,13,14]. This terminology emphasizes the hemodynamic nature of the configuration rather than a purely descriptive angiographic shape.

The angiographic “donut sign” [1] reflects wall-adjacent circulation and indicates persistent mural flow with central absence of contrast opacification. However, the “donut sign” is an imaging descriptor only, and may also be observed in certain serpentine aneurysms without implying the same underlying mechanism. In contrast, the ring-like aneurysm represents a dynamic flow-dependent state in which a focused, tangential inflow jet circulates along the aneurysm wall, maintaining a patent mural ring and preventing complete thrombosis [1,5,6].

### 4.1. Hemodynamic Interpretation

**Figure 1:**
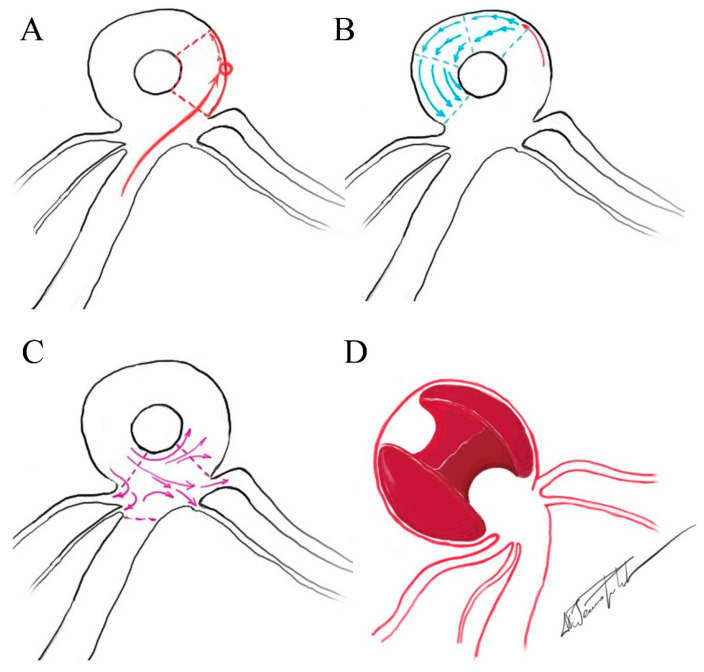
(**A**–**C**) *Schematic illustration demonstrating the flow dynamics within a bifurcation type ring-like aneurysm.* (**A**) Inflow jet zone—A focused inflow jet enters the sac and strikes the aneurysmal wall at a defined impaction point (red circle), after which the flow spreads tangentially along the wall. (**B**) Circumferential flow zone—Following the initial impact, blood travels within the peripheral mural channel, progressively decelerating as it courses toward the outflow region. (**C**) Neck zone—A complex region where circulating blood partially enters the daughter branches of the bifurcation, partially returns into the mural ring to recirculate, and partially collides with the incoming inflow jet, generating local turbulence. (**D**) *Schematic depiction of the expected intrasaccular thrombus morphology*—an “eaten apple–core” configuration, showing a central thrombus with concave surfaces molded by the adjacent mural flow channel.

**Figure 2:**
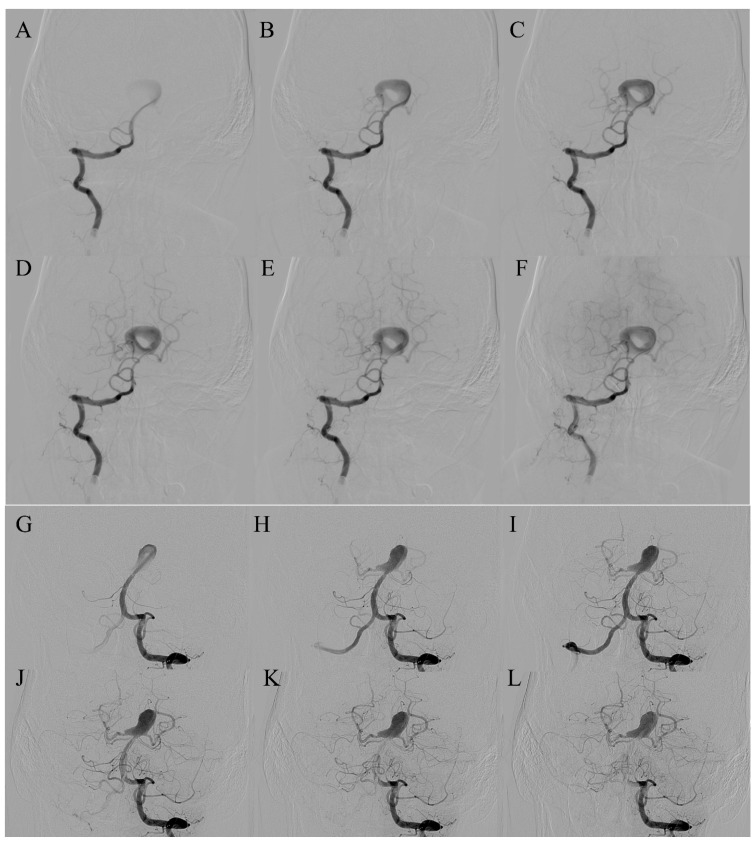
(**A**–**F**). *AP projection. Ring-like aneurysm at the tip of the basilar artery.* In the early arterial phases (**A**,**B**), a focused jet-inflow enters the aneurysmal sac, producing peripheral opacification with a central non-opacified core, consistent with shear-driven mural circulation around a thrombus. In the subsequent phases (**C**–**F**), there is progressive circumferential enhancement of the mural ring, while both ACP demonstrate delayed and attenuated opacification, reflecting prolonged intra-aneurysmal recirculation and slow outflow characteristic of the ring-like pattern. (**G**–**L**). *AP projection, same patient, six-week follow-up.* At follow-up, the aneurysm exhibits marked morphologic and hemodynamic evolution. The sac shows complete contrast opacification without a central “donut” defect (**G**–**I**), indicating collapse of the mural ring and loss of the previous ring-like configuration. Branches of the basilar artery now appear earlier and more distinctly opacified, confirming improved outflow and resolution of prior stagnation. This pattern is consistent with dynamic remodeling of the thrombus core and altered intra-aneurysmal flow after the initial angiographic study.

**Figure 3:**
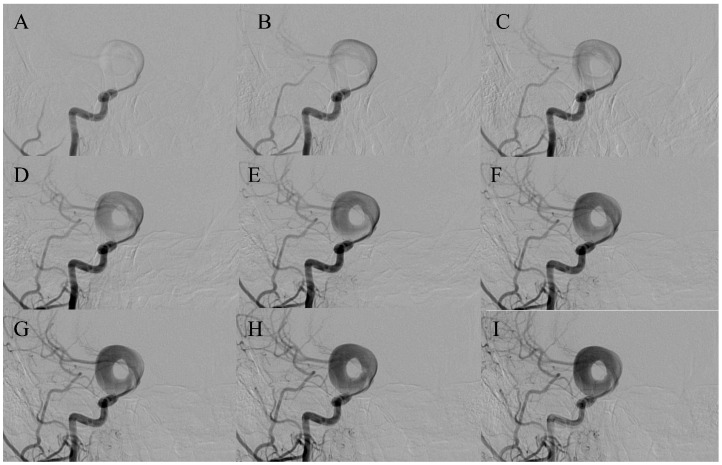
(**A**–**I**). *Ring-like aneurysm of the right ICA (C6 segment) demonstrating progressive circumferential opacification of the mural ring*. A focused and angulated jet-inflow enters the aneurysmal sac through a pre-aneurysmal narrowing, producing initial peripheral filling followed by complete circumferential enhancement of the mural channel. A central non-opacified zone corresponds to stagnant blood/thrombus core. The distal ICA and M1 segments show delayed and initially faint opacification, which becomes more intense in the late arterial phases as contrast egresses from the aneurysmal sac, consistent with prolonged intra-aneurysmal circulation characteristic of the ring-like pattern.

In the ring-like pattern aneurysms, shear-driven recirculation maintains flow along the mural ring while the central zone remains stagnant, causing gradual thrombus formation. Within its lumen, the flow architecture is tri-phasic, consisting of a focused inflow jet that impinges on the wall, a circumferential wall-adjacent recirculation zone that sustains the mural ring, and a progressively dispersed outflow stream that circulates towards the shared neck (Figure 1A). This hemodynamic arrangement may be consistent with the high rupture rate (37.5%) observed in pooled cases. To date, no prior publication has proposed a morphologic subdivision of ring-like aneurysms. We propose two reproducible patterns—bifurcation-type (Figure 2) and trunk-type (Figure 3)—each of which appears to be associated with characteristic circulation dynamics determined by the presence or absence of branching vessels. In trunk-type ring-like aneurysms, the lack of daughter branches creates a single inflow–outflow pathway through the common neck. Without lateral outflow, the contrast column remains within the sac for longer, resulting in more sustained intra-aneurysmal circulation and more pronounced flow complexity at the neck.

From a rheological perspective, blood within the ring-like lumen exhibits non-Newtonian behavior, where viscosity increases in low-shear regions and decreases in the high-shear mural layer. This shear-thinning property contributes to the maintenance of the peripheral jet, promoting persistent recirculation along the aneurysm wall while the central zone becomes progressively stagnant. The resulting architecture functionally forms a dual-compartment system—an outer, low-viscosity shear-maintained ring and an inner, high-viscosity thrombus core—forming a quasi-stable yet rupture-prone equilibrium [13,14]. Across the merged dataset, ring-like aneurysms show a markedly elevated rupture incidence compared with annual population estimates (1.6–6.5%) and even exceeding expected lifetime rupture risk (~30%). This may reflect a combination of larger aneurysm size, unique flow geometry, and case selection bias, but reinforces the distinctly high rupture potential that warrants clinical vigilance [15,16].

### 4.2. Dynamic Evolution of the Ring-like Configuration

**Figure 4:**
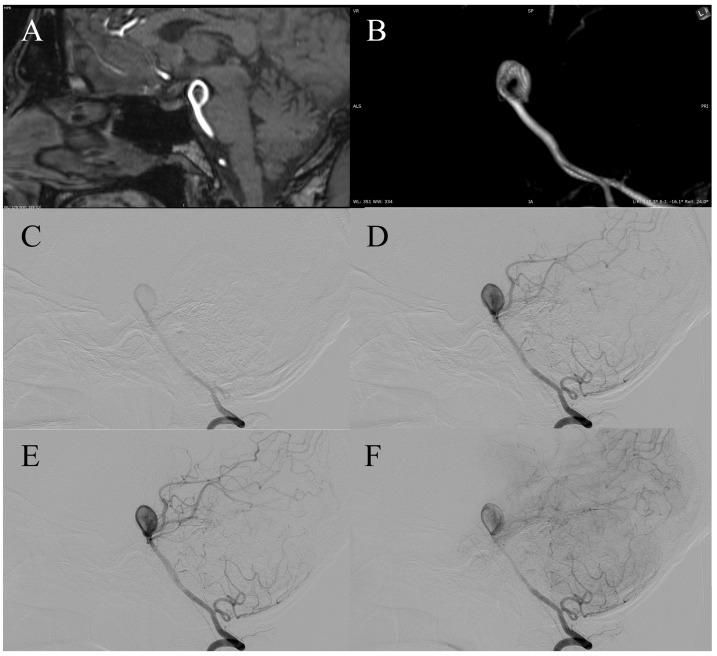
*Multimodal imaging of a dynamically evolving aneurysm of the basilar tip.* (**A**) 3D-TOF MRA (MIP reconstruction) shows a ring-like flow lumen with a well-defined circumferential flow channel surrounding a central flow-void, consistent with a partially thrombosed aneurysm with a donut-like configuration. (**B**) 3D-TOF MRA (VR reconstruction) clearly depicts the patent peripheral flow ring encircling the central thrombus, demonstrating a classic donut-sign. (**C**–**F**) Preprocedural digital subtraction angiography (early to late arterial phases) no longer demonstrates the ring-like configuration. Instead, the aneurysm fills homogeneously, presenting as a typical basilar tip aneurysm without a visible central non-opacified core. A persistent inflow jet through the aneurysmal neck is still evident, but the central thrombus seen on MRA is not well visualized on DSA, likely due to partial thrombus degradation, resulting in loss of the donut-sign.

The ring-like lumen is not a definitive anatomic subtype but a flow-dependent configuration sustained by shear-driven mural circulation. The “eaten apple-core” configuration of the central thrombus places its basal surfaces in direct contact with the aneurysm wall, exposing these regions to the same inflammatory, proteolytic, and hypoxic injury mechanisms described in large and giant partially thrombosed aneurysms [17,18,19,20]. In ring-like aneurysms, this mural weakening can further be compounded by the circumferential jet-inflow that courses along the wall, creating a dual pattern of biochemical and shear-related stress. The dynamic nature of this configuration is evident in both published and original cases. In the first case of the series by van Rooij et al. [2], a ring-like pattern emerged two weeks after stent implantation, indicating that altered inflow geometry and peripheralization of the jet can create a circumferential flow channel even when it is not initially present. In contrast, in Case 4 of our series [10], a clearly defined ring-like aneurysm was confirmed on diagnostic DSA, yet six weeks later, at the time of planned embolization, the entire outflow channel was no longer angiographically visible (Figure 2G–L). Case 5 further supports the dynamic nature of this morphology. On pre-procedural MRA, the aneurysm demonstrated a classic ring-like configuration with a central signal void corresponding to organized thrombus. However, diagnostic DSA performed several weeks later revealed almost complete recanalization of the aneurysmal sac, with faint residual central contrast opacification (Figure 4). This transformation indicates near-complete dissolution of the central thrombus and re-establishment of mural flow, underscoring that the “ring-like” appearance may fluctuate over short time intervals depending on the balance between shear-driven flow and intraluminal thrombus stability. These contrasting transformations show that the configuration appears to be governed by instantaneous hemodynamic conditions rather than by stable structural features. Dynamic changes in thrombus morphology should be interpreted not as direct indicators of rupture risk, but as evidence of underlying hemodynamic instability—an element that may contribute to the symptomatic and rupture behavior observed in reported cases.

### 4.3. Histopathology and Biology

Histologic and 7T MRI studies have reported that partially thrombosed aneurysms harbor vasa vasorum proliferation, inflammation, and intramural hemorrhage adjacent to the thrombus [12,14]. These biological processes weaken the wall, particularly in regions exposed to shear stress. The mural lumen in partially thrombosed aneurysms is therefore not inert, but a biologically active interface capable of enzymatic degradation and progressive wall thinning.

### 4.4. Clinical Implications

Radiologically, the ring-like configuration may be mistaken for stable thrombosis, but it should be interpreted as a hemodynamic warning. Cases in both adults and children confirm that ring-like aneurysms may rupture without warning [1,13]. Flow-diverter reconstruction represents a physiologically plausible approach: by suppressing tangential inflow and modifying wall-shear distribution, the device promotes gradual thrombosis and healing [7]. However, transient perianeurysmal edema may occur post-treatment, likely reflecting wall inflammation and thrombus reorganization [8,9,19,21,22,23]. In some instances, delayed rupture may develop either spontaneously or after incomplete hemodynamic exclusion, which may reflect insufficient thrombosis of the mural ring under persistent inflow stress [2,5]. These observations underscore the potential importance of early and complete endovascular reconstruction of the inflow zone.

### 4.5. Comparison with Serpentine Aneurysms

Serpentine aneurysms fundamentally differ in their flow architecture, which features separate inflow and outflow tracts and characteristically sluggish intra-aneurysmal flow. In contrast, ring-like aneurysms maintain a single neck with a focused, mural inflow jet—a configuration that, based on available case reports, appears to confer reduced stability and a higher propensity for rupture [1,2,4,7,14].

### 4.6. Demography and Localization

The female predominance observed in this pooled cohort mirrors the established gender distribution of intracranial aneurysms in general and therefore does not appear to be specific to the ring-like subtype. Ring-like aneurysms most frequently occur in the supraclinoid ICA and at the basilar apex-regions characterized by complex flow curvature and branching that may predispose to persistent mural ring flow [1,2,4,7,8,24].

### 4.7. Future Perspectives

Future work should combine patient-specific CFD with serial MRI and histologic correlation to better characterize shear-stress conditions associated with rupture risk [25]. Dedicated CFD simulations of ring-like aneurysms are warranted to characterize shear-stress heterogeneity within the mural ring and to identify hemodynamic patterns that may precede or contribute to rupture. Such modeling could help bridge the current gap between morphologic observation and quantitative hemodynamic analysis. Establishing a prospective registry of ring-like aneurysms may further clarify their natural history and treatment outcomes.

## 5. Conclusions

Ring-like aneurysms represent a reproducible and high-risk morphologic subtype within the spectrum of partially thrombosed intracranial aneurysms. The pooled rupture prevalence of 37.5% suggests that this configuration reflects an actively maintained, shear-dependent state rather than an end-stage thrombotic residue. The neuroradiologic “donut sign” may serve as a potential imaging indicator of an underlying jet-inflow pattern, reflecting persistent mural circulation and an active hemodynamic state associated with increased rupture risk. Early identification of this morphology and timely flow-modifying treatment may be important in preventing progression to rupture.

## Figures and Tables

**Table 1:** Individual Published Cases of “Ring-like” (Donut-shaped) Intracranial Aneurysms (1996–2024, PubMed-indexed). BA—basilar artery; ICA—internal carotid artery; C7—communicating segment; C6—ophthalmic segment; C4—cavernous segment; SCA—superior cerebellar artery; AComA—anterior communicating artery; PComA—posterior communicating artery; MCA—middle cerebral artery; SAH—subarachnoid hemorrhage; ICH—intracerebral hemorrhage; IVH—intra-ventricular hemorrhage; TIA—transitory ischemic attack; C—coiling; SAC—stent assisted coiling; FDS—flow-diverting stent; WEB—woven endobridge device.

#	Author(s)	Year	Journal	Location	Size (mm/Type)	Sex/Age	Clinical Presentation	Treatment	Clinical Outcome	Paper Highlights
1	Ogawa, T. et al. [1]	1996	Am J Neuroradiol	BA tip	22 (Large)	F/56	No data (Compression of upper part of the pons)	No data	No data	First documented use of the term “doughnut-shape” in the description.
2	Takeuchi, S. et al. [4]	2012	Acta Neurol Belg	ICA-C6	15 (Large)	F/51	SAH	Trapping + A3–A3 bypass	Good	First use of term “doughnut-shaped aneurysm”.
3	van Rooij, S.B.J. et al. [2]	2014	Interventional Neuroradiology	BA tip	20 (Large)	F/50	Intermitted dizziness	SAC	Good	Introduction of term “donut-sign”; First stent induced case.
4	van Rooij, S.B.J. et al. [2]	2014	Interventional Neuroradiology	BA-SCA origin	18 (Large)	F/70	Repeated TIA	C	Good	
5	van Rooij, S.B.J. et al. [2]	2014	Interventional Neuroradiology	ICA tip	17 (Large)	M/46	SAH	SAC	Death	
6	Maingard, J. et al. [5]	2016	Interventional Neuroradiology	BA tip	20 (Large)	F/51	Symptomatic (headache)	FDS	Good	N/A
7	Cholet, C. et al. [6]	2017	World Neurosurg	AComA	27 (Giant)	M/30	SAH	Clipping	Death	N/A
8	Lee, C.H. et al. [7]	2017	Acta Neurochir	ICA–PComA	15 (Large)	M/59	SAH	Clipping	Good	First report to prove “donut-shape” is consequence of central thrombus; Different mechanisms of thrombus formation in doughnut-shaped and giant serpentine aneurysms; Fibrinoid-exudate in aneurysm sac.
9	Sgreccia, A. et al. [8]	2018	World Neurosurgery	ICA-C4	25 (Giant)	F/62	Retroorbital pain, visual impairment	FDS	Good	Elastic lamina degeneration, lack of muscular layer, recurrent subadventitial hemorrhages.
10	Vollherbst, D.F. et al. [9]	2019	Journal of Clinical Neuroscience	ICA–C6	27 (Giant)	F/49	Headaches	FDS + WEB + C	Good	Composite treatment.
11	Nestorović, D. et al. [10]	2024	Medicina (Kaunas)	MCA bifurcation	17 (Large)	F/59	SAH + ICH + IVH	Conservative	Death	Largest series; “Ring-aneurysm” configuration disruption; Smallest and largest aneurysm of this type described; “Eaten apple core” intrasaccular thrombus morphology.
12	Nestorović, D. et al. [10]	2024	Medicina (Kaunas)	ICA-C6/C7	27 (Giant)	F/33	Headaches, visual impairment	FDS	Good	
13	Nestorović, D. et al. [10]	2024	Medicina (Kaunas)	ICA-C4	36 (Giant)	F/51	Headaches, visual impairment	FDS	Good	
14	Nestorović, D. et al. [10]	2024	Medicina (Kaunas)	BA tip	25 (Giant)	F/48	Headaches, vertigo	SAC	Good	
15	Nestorović, D. et al. [10]	2024	Medicina (Kaunas)	BA tip	14 (Large)	M/46	Headaches	pCONus2 + C	Good	
16	Demartini, Z., Jr. et al. [11]	2024	World Neurosurg	BA tip (pediatric)	16 (Large)	M/11	SAH	C (Balloon-assisted)	Fair	First described pediatric ring-like aneurysm.

## Data Availability

The original data presented in the study are openly available in PubMed indexed Papers which we analyzed.
